# A critical evaluation of loss of heterozygosity detected in tumor tissues, blood serum and bone marrow plasma from patients with breast cancer

**DOI:** 10.1186/bcr1772

**Published:** 2007-10-03

**Authors:** Heidi Schwarzenbach, Volkmar Müller, Cord Beeger, Miriam Gottberg, Nicole Stahmann, Klaus Pantel

**Affiliations:** 1 Institute of Tumor Biology, University Medical Center Hamburg-Eppendorf, Hamburg, Germany; 2 Clinic of Gynecology, University Medical Center Hamburg-Eppendorf, Hamburg, Germany

## Abstract

**Introduction:**

The aim of the study was to perform a comparative analysis of LOH (loss of heterozygosity) in primary tumors as well as peripheral blood and bone marrow (BM) of patients with breast cancer (BCa).

**Methods:**

Performing PCR-based fluorescence microsatellite analysis and using a panel of seven polymorphic microsatellite markers, we compared the profiles of LOH in primary tumors, peripheral blood and BM plasma from patients with primary BCa (*n *= 40, stage M0) as well as tumor tissues and blood serum from metastatic BCa patients (*n *= 48, stage M1). During the course of systemic treatment blood samplings from 12 M0 and 16 M1 patients were at least once repeated.

**Results:**

The overall incidences of LOH in tumor tissues, blood and BM samples were 27.5%, 9.0% and 5.0%, respectively. The marker D3S1255 was the only one in the panel that showed similar frequencies of LOH ranging from 19.0 to 24.5% in tumor, blood and BM samples. Both M0 blood serum and BM plasma samples displayed the same rate of 19.0%, whereas tumor and M1 serum showed a rate of 24.5% and 24.0%, respectively, at this locus. This marker also showed the highest frequency of LOH in serum and BM samples, whereas in tumor samples LOHs at the markers D13S218 (38%) and D17S855 (36%) were more frequent. Statistical analysis of the tumor samples showed that occurrence of LOH at the markers D3S1255 (*P *< 0.04), D9S171 (*P *< 0.05) and D17S855 (*P *< 0.03) correlated with undifferentiated nuclear grade. Additionally, significant associations of the number of LOH recorded at D17S250 with estrogen receptor (*P *< 0.02), progesterone receptor (*P *< 0.03) expression and high proliferation score (Ki-67 expression, *P *= 0.009) were observed. In blood serum samples a relationship between positive lymph node status and LOH at the marker D3S1255 was revealed (M0 stage, *P *= 0.05; M0+M1 stage, *P *= 0.004).

**Conclusion:**

Our study demonstrates heterogeneous profiles and low rates of LOH, particularly on free DNA in BM and blood samples. However, the significant associations of LOH with some risk factors and the demonstrated possibility of monitoring free DNA in patients undergoing systemic therapy suggest that LOH analysis may be developed into a useful diagnostic tool.

## Introduction

Breast cancer (BCa) is the most frequent malignant cancer of women western industrial nations. One in ten women will develop BCa.

With the use of traditional prognostic factors, it has still not been possible to reliably identify those patients with BCa who will relapse with metastatic disease. Therefore, improved approaches should be developed to identify additional factors that enable individual risk assessment. Besides the classical factors being engaged in differentiation status, histological type, angio-invasion, steroid hormone receptor status, as well as the involvement of lymph nodes and age of patients with breast cancer, new prognostic and predictive factors are used. They reflect the morphological and molecular characteristics of tumors, such as S-phase-fraction, Ki-67, epidermal growth factor receptor (EGFR), *HER2 *oncogene, invasion and metastasis markers like tumor-associated proteases and associated inhibitors, and measurement of vessel density for the assessment of angiogenesis [1]. However, despite this multifactoral prognostic model and advances in the treatment of BCa, comprehensive knowledge of the genetic bases and biological consequences is also required. In addition, the established prognostic factors are determined in primary tumors that are routinely removed by surgery. Therefore, the development of blood or bone marrow (BM) tests with clinical relevance for risk estimation and measurement of residual tumor load is of great interest [2,3].

The carcinogenesis of breast cancer is a complex multistep process associated with numerous genetic alterations and an early hematogeneous dissemination of tumor cells [2-4]. DNA alterations and downregulation of tumor-suppressor genes, such as genes that encode molecules involved in cell adhesion, cell-cycle arrest and apoptosis, are associated with BCa [5]. Experimental studies introducing chromosomal DNA with deleted regions into cell lines have demonstrated that tumor growth and disease progression of BCa was promoted by such an introduction. The systematic use of microcell hybrid clones has identified a number of chromosomal regions that are important for the proliferation of breast tumor cells [6,7].

With different methods, such as microsatellite analyses and CGH (comparative genomic hybridization), a number of putative tumor-suppressor genes can be characterized [8-11]. Presence of allelic imbalance at definite chromosomal regions is sufficient to provide selective growth advantage of BCa cells. However, the number of known candidate genes that have a role in the development of BCa is still limited [12]. Among these genes, the recently identified tumor-suppressor genes *BRCA1 *and *BRCA2 *contribute to a substantial portion of inherited BCa [13]. During progression of BCa a potential relationship between specific genetic alterations at the locus 17q21 and hormonal deregulation has been observed [14]. Genetic aberrations that affect chromosome arms 7p, 16q, 17p and 17q seem to be early events [11]. LOH at region 16q22.2-q23.2 seems to be a major event in the genesis of metastases [15].

During tumorigenesis free DNA may be released early from apoptotic or necrotic cells into the blood circulation [16-18]. In the blood of patients with prostate cancer we detected increased concentrations of extracellular DNA [19]. Furthermore, allelic imbalance could be identified in the serum or plasma of patients with various tumor types [20-27]. Blood may therefore be a convenient source for the detection of such tumor-associated DNA abnormalities. It constitutes a pool of free DNA derived from different sources, amongst others from heterogenous areas of the primary tumor. A further important feature is the possibility of taking repeated blood samples during treatment.

Using PCR-based microsatellite analysis, our study demonstrates heterogeneous distribution of LOH in primary tumor, serum and BM of BCa patients and significant associations with established risk factors. To our knowledge, this is the first study to compare the presence and validity of LOH in different tissue compartments.

## Materials and methods

### Patients and samples

Blood serum of BCa patients with primary breast cancer (*n *= 40, stage M0) was taken after surgery before the initiation of adjuvant therapy. Blood serum of patients with metastatic disease (*n *= 48, stage M1) was collected 1 to 11 years after surgery of the primary tumor. Of 28 patients (*n *= 12 M0, *n *= 16 M1), repeated blood collections during the course of treatment were included. In addition, primary tumors and BM aspirates were available from 60 (*n *= 30 M0, *n *= 30 M1) and 22 patients (*n *= 22 M0), respectively. Blood samples were taken between February 2002 and October 2003 from patients who received systemic therapy at the outpatient clinic of the department of gynecology and who agreed to participate in this study.

Patients treated for primary BCa underwent surgery including lumpectomy and dissection of axillary lymph nodes or modified radical mastectomy. All patients had epithelial BCa, and metastatic spread was excluded by chest radiology, liver ultrasound scan and bone scan. These patients received adjuvant treatment (endocrine treatment and/or anthracycline-containing chemotherapy as well as radiotherapy) according to national guidelines.

Patients with metastatic disease received chemotherapy, endocrine treatment, or treatment with the humanized anti-HER2 antibody Trastuzumab (Herceptin) alone or in combination with chemotherapy.

The median age of M0 and M1 patients was 58 (range 29 to 77) and 59 (range 31 to 83) years, respectively.

All patients gave their informed consent and the examination of the clinical material was approved by the local ethics review board.

### Immunohistochemistry and DNA preparation from paraffin-embedded tissues

Sections of 5 μm thickness were cut from formalin-fixed, paraffin-embedded breast tumor blocks and mounted on microscope slides.

Paraffin-embedded sections of the primary tumors were stained for the expression of HER2 (antibody CB 11; Novocastra, Newcastle upon Tyne, UK), estrogen receptor (antibody NCL-6F11, Novocastra), progesterone receptor (antibody NCL-PGR-312, Novocastra) and the proliferation antigen Ki-67 (clone MIB-1; Dako, Hamburg, Germany). Staining for HER2 was expressed as 'DAKO-Score' with values between 0 and 3+ (only 3+ was considered as positive); staining for estrogen and progesterone receptors was evaluated according to the system of Remmele and Stegner [28], and a score greater 1 was regarded as positive. Proliferation was evaluated as the percentage of cells stained positive with the antibody MIB-1.

For microscopic evaluation the paraffin slices were stained with haematoxylin and eosin (Merck, Darmstadt, Germany). The tumor tissue of paraffin slices were either scraped off from H/E-stained slides under a microscope or computer-assisted microdissected from unstained slides via laser pressure catapultation.

### Preparation of serum and leukocytes

Approximately 10 ml blood were drawn by vein puncture and centrifuged at 2,500 *g *for 10 min. The upper phase contained the blood serum, from which 2 to 3 ml was removed for the extraction and analysis of the circulating DNA. The remaining 17 to 18 ml blood were supplemented up to 50 ml with lysis buffer containing 0.3 M sucrose, 10 mM Tris-HCl pH 7.5, 5 mM MgCl_2 _and 1% Triton ×100 (Sigma, Taufkirchen, Germany). Following incubation for 15 min on ice, the isolation and purification of the leukocytes were carried out by two centrifugation steps at 2500 *g*, 4°C for 20 min.

### Preparation of bone marrow specimens

BM specimens obtained in parallel with the blood samples were aspirated directly after surgery under general anaesthesia from both iliac crests and stored in heparinized tubes with DMEM (Gibco, Eggenstein, Germany). After centrifugation at 400 *g *for 5 min the supernatant containing the cell-free plasma for further analysis of circulating DNA was removed.

### DNA extraction and fluorencence-labeled PCR

Genomic DNA was extracted from microdissected tumor tissues, blood serum, BM plasma and leukocytes (reference DNA) of each patient using the QIAamp DNA Mini Kit and a vacuum chamber (Qiagen QIAvac24) according to the manufacturer instructions (Qiagen, Hilden, Germany). Quantifications and qualities of the isolated DNA were spectrophotometrically determined at 260 and 280 nm, respectively (Eppendorf, Hamburg, Germany). Dilution experiments to determine the lowest portion of tumor-specific DNA that could be detected were performed. For this study we mixed and amplified known quantities and proportions of normal (leukocyte) DNA and tumor or plasma DNA.

10 ng of DNA samples were amplified in a 10-μl reaction volume containing PCR Gold buffer (150 mM Tris-HCl, pH 8.0 and 500 mM KCl), 2.5 mM MgCl_2 _(Applied Biosystems, Mannheim, Germany) 200 μM dNTPs (Roche, Mannheim, Germany), 11 pM of primer sets (Sigma, Taufkirchen, Germany) and 1.5 U AmpliTaq Gold DNA-Polymerase (Applied Biosystems). The sense primers were fluorescence-labeled (Hex, FAM or TAMRA) at the 5'end. The reaction was started with activation of the DNA polymerase for 5 min at 95°C followed by 40 cycles of PCR amplification. To verify the microsatellite alterations, the experiments were done as duplicates and each PCR was repeated at least twice.

We chose the following microsatellite primers for the current analyses: D3S1255, D9S171, D10S1765, D13S218, D16S421, D17S250 and D17S855, because they are most informative for the particular gene loci and binding to known tumor-suppressor genes [12-16].

### Evaluation of the data

0.5 μl of each PCR product was mixed with 40 μl HiDi Formamid and 0.2 μl 500-ROX size marker (Applied Biosystems, Darmstadt, Germany), which served as an internal standard. Each sample was denatured by heating to 94°C for 2 min. Capillary electrophoresis analysis was performed on a Genetic Analyzer 310 (Applied Biosystems). Samples were injected at 15 kV for 3 s and separation was performed at 15 kV and 60°C. Data were collected with data-collection software and analyzed by Gene-Scan 3.1 software. GeneScan-500 standard fragments in the size range of 100 to 300 bp were used for the calculation of relative sizes of microsatellite alleles. The incidence of LOH was calculated by the ratio of the intensities of the two alleles in tumor tissue, serum and BM plasma in compared with leukocytes. LOH was interpreted if the final quotient was < 0.6 or >1.67. Homozygous and non-analyzable peaks were designated as non-informative cases.

### Statistical analysis

The statistical analysis was performed using the SPSS software package, version 13.0 (SPSS Inc., Chicago, Illinois, USA). Fischer's exact test was used to identify possible associations of the pattern of LOH with the clinical parameters, such as tumor stage (TNM), nuclear grade, estrogene receptor, progesterone receptor, HER score and the proliferation antigen Ki-67. A value of *P *≤ 0.05 was considered as statistically significant.

## Results

### Comparison of yields of serum DNA from BCa patients and healthy women

The concentration of DNA in serum of 88 BCa patients was spectrophotometrically quantified, and revealed a range of DNA yields between 70 and 5,000 ng/ml, with a mean value of 500 ng/ml. There was no difference in concentrations of serum DNA between stages M0 and M1. The range of DNA concentrations in the 22 BM samples was between 80 and 800 ng/ml with a mean value of 310 ng/ml, and consequently lower than the DNA levels in serum. Compared with the higher DNA concentrations in blood and BM plasma of BCa patients, healthy women had minor levels of DNA in their serum. The mean value of the DNA concentrations of serum from 10 control women was 63 ng/ml. Repeated blood collections during 3 months showed a constantly low level of DNA in these samples and no occurrence of LOH on the free DNA (data not shown).

### Frequency of LOH in primary tumor, blood serum and BM plasma

**Figure 1:**
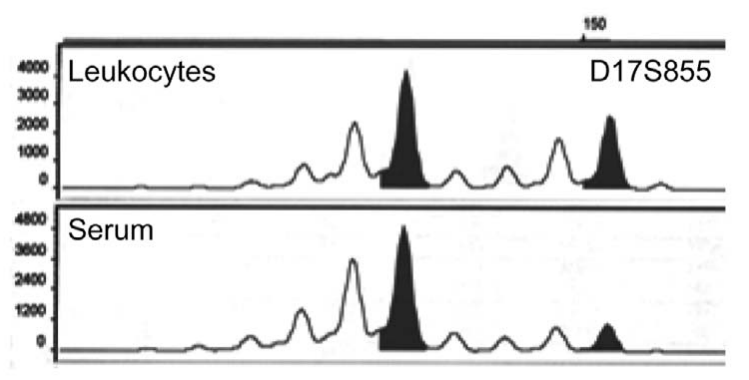
Example of a loss of heterozygosity (LOH) detected in blood serum from a patient with breast cancer. The fluorescence-labeled PCR products of leukocytes and serum DNA were separated by capillary gel electrophoresis on a Genetic Analyzer and evaluated with the Gene Scan Analysis program. The abscissa indicates the length of the PCR product, while the ordinate gives information on the fluorescence intensity represented as peaks. The upper and lower diagram show the leukocyte DNA (reference) and serum DNA amplified with the primer binding at the D17S855. The PCR product of the serum DNA shows a LOH.

Additional file 1 outlines the pattern of LOH detected in primary tumors, blood and BM of each BCa patient with primary (*n *= 40, stage M0) and metastatic disease (*n *= 48, stage M1) using a panel of 7 different polymorphic microsatellite markers. Blood serum of the 40 M0 patients was taken after removal of the primary tumor.' Tumor tissues and BM aspirates were available from 30 and 22 of these patients, respectively. Primary tumors from 30 of the 48 M1 patients were obtained at the time of primary surgery, when these patients displayed no clinical signs of overt metastases (that is, 1–11 years before blood sampling). As shown in Additional file 2, 30% of the BCa patients (M0 and M1) harbored at least one LOH at any of the markers tested in their blood serum. Figure 1 shows such a LOH on free serum DNA recorded at the marker D17S855. By contrast, 60% of the patients displayed LOH in their primary tumor, while LOH was detected in the BM plasma in only 18% of these patients.

**Figure 2:**
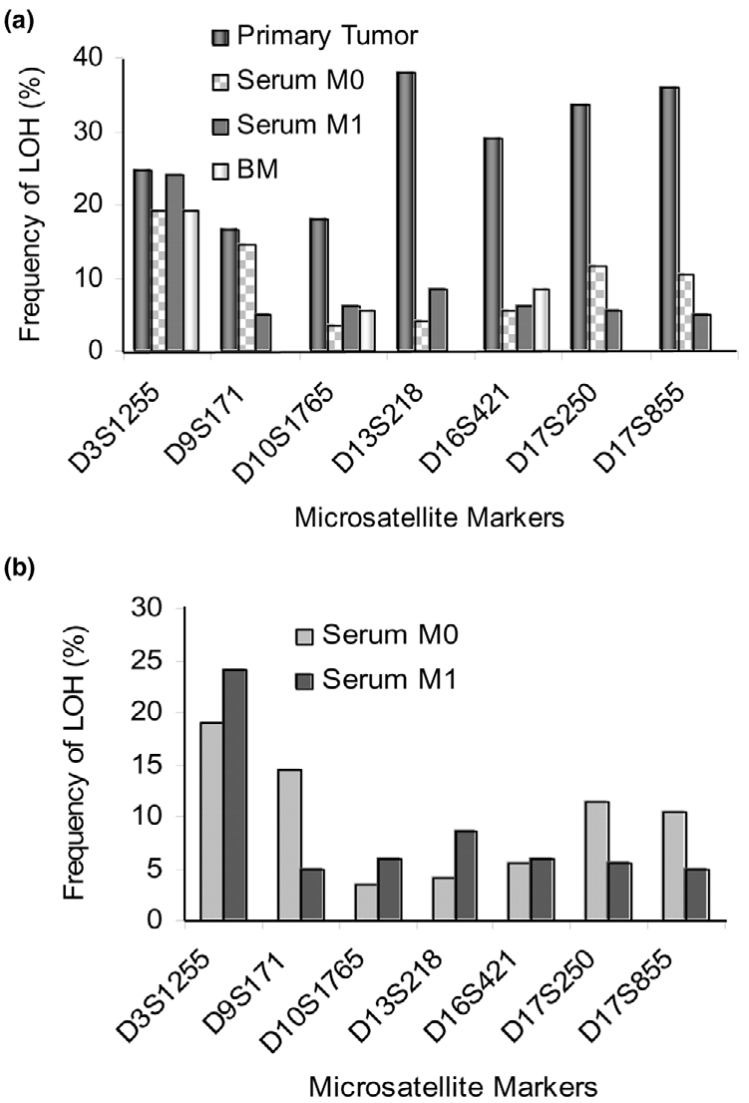
Comparison of the frequency of loss of heterozygosity (LOH) at seven different polymorphic microsatellite markers detected in tumor tissues, blood and bone marrow samples from patients with primary (M0) and metastatic (M1) breast cancer. The frequency of LOH was calculated by division of the number of LOHs with the informative cases for each locus.

The LOH index (number of LOH of each patient divided by the informative cases) of the respective patients was higher in primary tumors (27.5%) than in serum (9.0%) and BM (5.0%) samples. Almost half (47%) of the LOHs at any of the markers recorded in serum could be retrieved in the corresponding primary tumors. The detected LOHs in BM could be recovered in 40% and 20% of the matched tumor and blood samples, respectively. Among the informative cases, the frequency of LOH in the tumors was highest for the markers D13S218 (38.0%), D17S855 (36.0%) and D17S250 (33.5%) (Figure 2a). The marker D3S1255 was the predominantly affected region in M0 (19%) and M1 (24%) blood serum, as well as in BM plasma (19%), indicating the same incidence of LOH on serum and BM DNA from M0 patients and a similar rate in the other clinical samples (Figure 2a,b). The high frequencies of LOH at the markers D10S1765 (18.0%), D13S218 (38.0%), D16S421 (29.0%), D17S250 (33.5%) and D17S855 (36.0%) found in tumors could not be retrieved in M0 serum samples (Figure 2a). A comparison of the numbers of LOH recorded in both sera from M0 and M1 patients in Figure 2b shows the increase of the rate of LOH at the markers D3S1255, D10S1765, D13S218 and D16S421 from M0 to M1 stage. At the other markers (D9S171, D17S250 and S17S855) the frequency of LOH was even higher in M0 than M1 serum. The overall rate of LOH in M0 and M1 serum were 10.0% and 8.5%, respectively.

### LOH profiles in serial serum samples during the course of adjuvant chemotherapy and treatment

To obtain information on the frequency of LOH during the course of adjuvant chemotherapy and treatment, serial examinations were performed using 1–3 repeated serum samples from 28 BCa patients (*n *= 12 M0, *n *= 16 M1). These patients were randomly chosen from the entire BCa patient cohort. In general, the patterns of the repeated blood collections showed no significant differences (Additional file 3).

Of the M0 patients, two women showed LOH in their first serum sample that which disappeared during the adjuvant therapy. By contrast, in serum of two further M0 patients with an initial LOH-negative result, LOH could only be observed in the second and third serum samples (Additional file 3).

The M1 serum samples displayed more frequent LOH during treatment than M0 serum. Whereas two women of the cohort showed LOH in their first serum sample which disappeared during therapy, four patients were initially LOH-negative, and LOH was only detected during therapy in the second and/or third serum samples (Additional file 3).

### Statistical evaluations of the LOH profiles with the established risk parameters

To determine whether a correlation exists between clinical parameters of the BCa patients and LOHs detected at the different microsatellite markers, we compared tumor size (T1, T2, T3/4), nuclear grade (G1, G2, G3), axillary lymph node status (positive/negative), estrogen receptor (positive/negative), progesterone receptor (positive/negative), the proliferation antigen Ki-67 (low ≤30%/high > 30%) and HER2 expression (positive/negative) with the occurrence of LOH in the different clinical samples. In Additional file 4, the statistically significant associations of LOH profiles detected in primary tumors and blood serum samples with the clinical data are summarized. In the tumor samples, several significant correlations could be detected. The occurrence of LOH at three markers (D3S1255, D9S171 and D17S855) was significantly associated with higher nuclear grade of tumors. At the marker D17S250 LOH was associated with negative estrogen and progesterone receptor status and the high expression of the proliferation antigen Ki-67. Besides its association with the nuclear grade, the marker D9S171 also showed a correlation with the antigen Ki-67 (Additional file 4). Interestingly, in M0 blood serum samples a correlation of LOH at the locus D3S1255 to positive axillary lymph node status was observed (Additional file 4, *P *= 0.05). Only M0 patients with a positive lymph node status harbored LOHs at this marker. Also, both M0 and M1 blood serum samples together showed a significant correlation of positive axillary lymph node status to LOH at this marker (Additional file 4, *P *= 0.004).

A statistical evaluation of the BM samples was not performed because of the small number of BM samples (*n *= 22) and the modest percentage of LOH detected in these samples (5%).

## Discussion

Our present microstallite study describes the detection of free tumor-associated DNA in blood serum and BM plasma of BCa patients with primary and metastatic disease, and the comparison of the obtained data with the LOH profiles analyzed in the matched primary tumors. In contrast to most recent publications that apply a similar molecular approach but only examine single compartments, we compared the coincidence of LOH in different tissues. Our panel of seven polymorphic microsatellite markers showed a higher LOH index in primary tumors than in serum and BM samples. The implication of our finding indicates that apart from free tumor-associated DNA blood of BCa patients may also contain normal DNA. The prevalence of normal DNA and the large dilution of free tumor DNA by normal DNA may hamper detection of genetic alterations in blood as discussed by several laboratories for other tumor entities [29-32]. However, the rates of LOH detected in these specimens are similar to the range seen in other studies [11,14,15].

Surprisingly, when we compared the rate of LOH in blood from BCa patients with primary and metastatic disease, we noticed a higher frequency of LOH in blood derived from M0 patients than in blood from M1 patients, suggesting that DNA loss may contribute to early breast tumorigenesis. Another reason for the lower incidence of LOH in M1 serum could be that metastatic patients received treatment. In addition, the elimination of the primary tumor may lead to the clearance of tumor-associated DNA. Moreover, the detection of tumor-associated DNA in blood from M0 patients after the removal of their primary tumor and also during the course of adjuvant treatment may be explained by the persistence of tumor cells also observed in blood and BM [33,34]. In line with this observation, the presence of LOH was not associated with tumor size, indicating that factors other than the tumor load influence the presence of such DNA in serum. The fact that free tumor DNA still circulates in M1 blood sustains the theory that tumor DNA is not only a signature of apoptotic or necrotic cells from the primary tumor, but also of those from metastatic sources [35]. The combination of free tumor DNA from different sources therefore makes blood an opportune clinical material for the analysis of LOH.

With the exception of the polymorphic marker D3S1255, we could observe a heterogeneous distribution of LOH in primary tumor, serum and BM plasma. The pattern of LOH at the marker D13S1255 was relatively uniform in tumor tissue, serum and BM samples. By contrast, the lack of reflection of the genetic patterns at the other markers in the primary tumor, M0 blood and BM may correspond to the known multifocal heterogeneity of these tumors. Furthermore, the possible integration of tumor DNA derived from micrometastatic cells should also be considered [22]. The discordant profiles of LOH in the primary tumor and M1 blood could be due to the different intervals between the sampling of serum from M1 patients and surgery of the primary tumor, and possibly tumor DNA released from metastasis [36]. Furthermore, the microsatellite markers seem to be limited by their sensitivity in serum or plasma. The use of SNP (single nucleotide polymorphism) markers that only differ from each other at a single nucleotide has recently been described to provide reliable and high quality data on a range of different DNA templates and to display a higher sensitivity than the classical microsatellite markers that exhibit length polymorphism. Further studies will be required to investigate whether LOH at SNP markers are more easily detectable in blood than LOH at microsatellite markers [37,38].

A number of previous studies have addressed LOH mapping in the blood or BM plasma of breast cancer patients, and have shown the prognostic importance of circulating microsatellite markers [20,23,39]. In addition, there are several publications describing infrequent occurrence and non-concordance of LOH in blood to the paired tumors [22,29-32]. To begin to clarify these discrepancies we additionally determined the profile of LOH during a follow-up study. The overall pattern of the serial serum DNA samples was largely consistent. Whether the detected non-concordant results are due to a changed clinical status is unclear. Also, we cannot rule out the possibility of a shift of the relative proportions between tumor-specific and normal DNA.

The occurrence of LOH at the marker D3S1255 in the serum of lymph-node-positive M0 patients but not in lymph-node-negative patients in our BCa cohort suggests that such a blood test may reflect tumor spread. An extended and combined selection of microsatellite markers could provide a better understanding of tumor progression in breast cancer and might facilitate the identification of high-risk breast cancer patients. Furthermore, the development of refined techniques for the extraction of free tumor DNA excluding particularly the proportion of normal DNA might advance the detection of LOH in blood. The significant association of the profile of LOH at the marker D3S1255 to the axillary lymph node status recorded in M0 and M0+M1 serum samples could not be detected in the tumor tissues. However, to obtain an accurate profile of genetic alterations in tumor tissues, the heterogeneity of breast tumors requires demanding analyses of several areas of the microdissected tissues. So far, there is little information on the microsatellite marker D3S1255 in the literature. The marker maps to the chromosomal locus 3p23 and encodes for arrhythmogenic right ventricular dysplasia 5 (ARVD5). A prognostic significance of LOH at 3p23 for patients with colorectal carcinoma has been reported [40]. In squamous cell carcinoma of the oral cavity and ovarian cancer, frequent DNA losses were observed at this region [41,42].

Our statistical evaluation of the data obtained from the primary tumors show significant associations between DNA deletions at several markers and the clinopathological parameters. Conversely, these significances could not be found in blood derived from M0 patients. We detected that the markers D3S1255, D9S171 and D17S855 significantly correlated with the nuclear grade of tumor cells. The frequent allele losses at the marker D9S171, which is located in the vicinity of the *CDKN2 *gene, whose gene product is a negative regulator of the cell cycle, was suggested to be involved in the pathogenesis of sporadic breast cancer [43]. The frequency of LOH at D9S171 was associated with the development of gastric cancer [44]. Concomitant losses of the *BRCA1 *region, which is located at the marker D17S855 together with the *BRCA2 *region were significantly related to the histological grade of the tumor [45]. Moreover, our current results denote a significant correlation of the marker D17S250, which is localized centromeric to *BRCA1 *gene, with the estrogen and progesterone content and the proliferation marker Ki-67. A highly significant correlation of the marker D17S250 with positive progesterone receptor status has been reported [46]. These significances only detected in primary tumors may be masked by the prevalence of normal DNA in blood.

## Conclusion

The significant associations of LOH with some risk factors and the demonstrated possibility to monitor free DNA in patients undergoing systemic therapy suggest that LOH analysis may be developed into a useful diagnostic tool. However, the low rate of LOH recorded in blood, the lack of concordance of the LOH patterns in the different tissues, and the small and coincidentally created set of microsatellite markers indicate that the method needs to be further improved. Therefore, refinement of the DNA extraction method to remove any excess of normal DNA that might conceal the tumor DNA should be aspired to. Examinations of larger and combined panels of different microsatellite markers in a multiplex PCR might improve the identification of DNA abnormalities, providing further insights into the complex pathogenesis of BCa.

## Abbreviations

BCa = breast cancer; BM = bone marrow; LOH = loss of heterozygosity.

## Competing interests

The authors declare that they have no competing interests.

## Authors' contributions

HS, CB and MG performed all experiments. HS performed the statistical analysis and drafted the manuscript and KP revised the manuscript. VM and NS prepared the clinical material and summarized the clinical parameters. HS, VM and KP were involved in conception and design of the study and participated in the discussion and interpretation of results.

## Supplementary Material

Additional file 1 Table showing a summary of loss of heterozygosity (LOH) and the incidence of LOH at seven different polymorphic markers in tumor, blood serum and bone marrow plasma of patients with primary (M0, *n *= 40) and metastatic (M1, *n *= 48) breast cancer. Click here for file

Additional file 2 Table showing the number of breast cancer patients and the incidence of loss of heterozygosity in their blood serum, tumor tissue and bone marrow plasma sample. Click here for file

Additional file 3 Table showing the overall incidence of loss of heterozygosity in repeated blood serum samples from 28 breast cancer patients (*n *= 12 M0, *n *= 16 M1) during the course of treatment. Click here for file

Additional file 4 Table showing the associations of loss of heterozygosity at the different markers recorded in tumor and M0 blood serum samples with established risk factors. Click here for file
