# Supplementary material for: A critical evaluation of loss of heterozygosity detected in tumor tissues, blood serum and bone marrow plasma from patients with breast cancer
Source: Breast Cancer Res. 2007 Oct 3;9(5):R66. doi: 10.1186/bcr1772 (PMC2242661; doi:10.1186/bcr1772)
Supplement: Additional file 1 — Table showing a summary of loss of heterozygosity (LOH) and the incidence of LOH at seven different polymorphic markers in tumor, blood serum and bone marrow plasma of patients with primary (M0, n = 40) and metastatic (M1, n = 48) breast cancer. [file bcr1772-S1.doc]

Table I Summary of LOH and the incidence of LOH at seven different polymorphic markers in tumor, blood serum and BM plasma of BCa patients with primary (M0, n=40) and metastatic (M1, n=48) disease

Total, number of all LOHs found in tumor (T), blood serum (S) and BM plasma samples.

LOH, retention of heterozygosity, non-informative and no clinical material.
