# Supplementary material for: A critical evaluation of loss of heterozygosity detected in tumor tissues, blood serum and bone marrow plasma from patients with breast cancer
Source: Breast Cancer Res. 2007 Oct 3;9(5):R66. doi: 10.1186/bcr1772 (PMC2242661; doi:10.1186/bcr1772)
Supplement: Additional file 2 — Table showing the number of breast cancer patients and the incidence of loss of heterozygosity in their blood serum, tumor tissue and bone marrow plasma sample. [file bcr1772-S2.doc]

Table II Number of BCa patients and the incidence of LOH in their blood serum, tumor tissue and BM plasma sample.
