# Supplementary material for: A critical evaluation of loss of heterozygosity detected in tumor tissues, blood serum and bone marrow plasma from patients with breast cancer
Source: Breast Cancer Res. 2007 Oct 3;9(5):R66. doi: 10.1186/bcr1772 (PMC2242661; doi:10.1186/bcr1772)
Supplement: Additional file 3 — Table showing the overall incidence of loss of heterozygosity in repeated blood serum samples from 28 breast cancer patients (n = 12 M0, n = 16 M1) during the course of treatment. [file bcr1772-S3.doc]

Table III Overall incidence of LOH in repeated blood serum samples from 28 BCa patients

(n=12 M0, n=16 M1) during the course of treatment.

1 LOH

2 LOH

retention of heterozygosity
