# Supplementary material for: A critical evaluation of loss of heterozygosity detected in tumor tissues, blood serum and bone marrow plasma from patients with breast cancer
Source: Breast Cancer Res. 2007 Oct 3;9(5):R66. doi: 10.1186/bcr1772 (PMC2242661; doi:10.1186/bcr1772)
Supplement: Additional file 4 — Table showing the associations of loss of heterozygosity at the different markers recorded in tumor and M0 blood serum samples with established risk factors. [file bcr1772-S4.doc]

Table IV Associations of LOH at the different markers recorded in tumor and M0 blood serum samples with established risk factors.

---, no significance detected

*M0, blood serum from M0 patients

^§^M0+M1, blood serum from M0 and M1 patients
